# Advanced imaging for risk stratification for ventricular arrhythmias and sudden cardiac death

**DOI:** 10.3389/fcvm.2022.884767

**Published:** 2022-08-22

**Authors:** Eric Xie, Eric Sung, Elie Saad, Natalia Trayanova, Katherine C. Wu, Jonathan Chrispin

**Affiliations:** ^1^Division of Cardiology, Department of Medicine, Section of Cardiac Electrophysiology, Johns Hopkins University School of Medicine, Baltimore, MD, United States; ^2^Department of Biomedical Engineering, Johns Hopkins University, Baltimore, MD, United States

**Keywords:** sudden cardiac death (SCD), ventricular arrhythmias, cardiovascular magnetic resonance (CMR), positron emission tomography (PET), single-photon emission computerized tomography (SPECT), computed tomography

## Abstract

Sudden cardiac death (SCD) is a leading cause of mortality, comprising approximately half of all deaths from cardiovascular disease. In the US, the majority of SCD (85%) occurs in patients with ischemic cardiomyopathy (ICM) and a subset in patients with non-ischemic cardiomyopathy (NICM), who tend to be younger and whose risk of mortality is less clearly delineated than in ischemic cardiomyopathies. The conventional means of SCD risk stratification has been the determination of the ejection fraction (EF), typically *via* echocardiography, which is currently a means of determining candidacy for primary prevention in the form of implantable cardiac defibrillators (ICDs). Advanced cardiac imaging methods such as cardiac magnetic resonance imaging (CMR), single-photon emission computerized tomography (SPECT) and positron emission tomography (PET), and computed tomography (CT) have emerged as promising and non-invasive means of risk stratification for sudden death through their characterization of the underlying myocardial substrate that predisposes to SCD. Late gadolinium enhancement (LGE) on CMR detects myocardial scar, which can inform ICD decision-making. Overall scar burden, region-specific scar burden, and scar heterogeneity have all been studied in risk stratification. PET and SPECT are nuclear methods that determine myocardial viability and innervation, as well as inflammation. CT can be used for assessment of myocardial fat and its association with reentrant circuits. Emerging methodologies include the development of “virtual hearts” using complex electrophysiologic modeling derived from CMR to attempt to predict arrhythmic susceptibility. Recent developments have paired novel machine learning (ML) algorithms with established imaging techniques to improve predictive performance. The use of advanced imaging to augment risk stratification for sudden death is increasingly well-established and may soon have an expanded role in clinical decision-making. ML could help shift this paradigm further by advancing variable discovery and data analysis.

## Introduction

Sudden cardiac death is an unexpected death from a cardiac cause within a short period (typically an hour or less) from symptom onset or, if unwitnessed, within 24 h of last being seen alive (1, 2). While the incidence of SCD has gradually declined over the past decades, the annual incidence is ~200,000–400,000 cases per year (the extensive range being attributable to the uncertainty of the cause of some deaths), amounting to around 15–20% of all deaths (3). Among patients with known cardiovascular disease (CVD), which represents about half of cases of SCD, ventricular arrhythmia (VA) is the leading mechanism of SCD in both ischemic cardiomyopathy (ICM) and non-ischemic cardiomyopathy (NICM). In ICM, obstructive coronary artery disease (CAD) leads to myocardial scarring, and regions of heterogeneous conduction serve as substrates for initiating ventricular tachycardia (VT). NICM encompasses diverse cardiac conditions that can result in scar formation or fibrosis, which along with electrophysiological remodeling, can result in VT or ventricular fibrillation (VF). The entity of ischemia but no CAD (INOCA) has also been described and remains an area of active investigation. Thus, far in clinical practice, primary prevention and risk reduction of SCD is accomplished with medical therapy and the implantable cardiac defibrillator (ICD), with slightly different approaches for patients with previously detected VA or resuscitated arrest. Guidelines for primary prevention ICD in both ICM and NICM are driven by clinical symptoms of heart failure and decreased ejection fraction (EF) <35% as derived by imaging (4). However, more useful prognostic data can be obtained from imaging than EF alone (5, 6).

**Figure 1:**
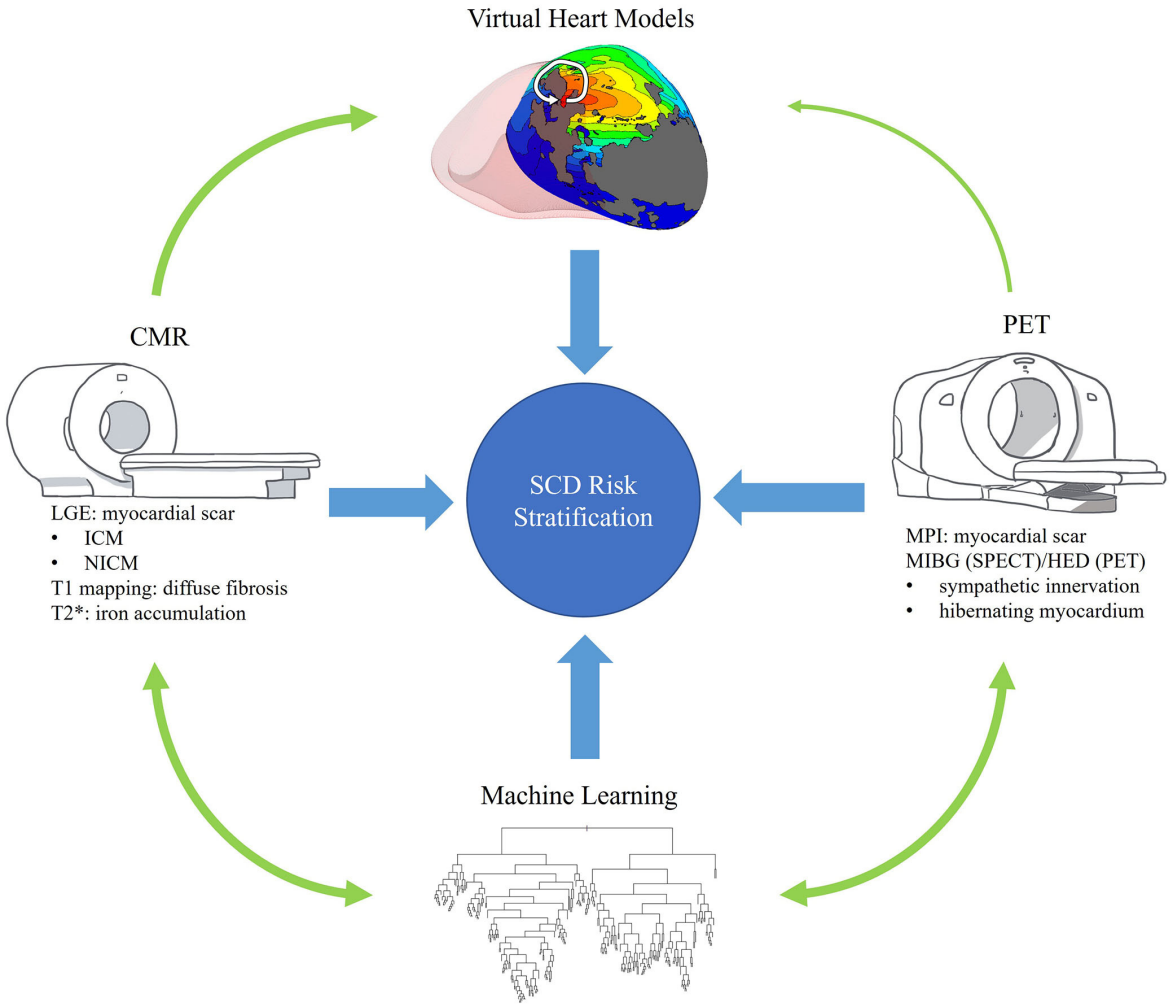
Overview. This review seeks to describe the role of advanced imaging in the risk stratification of SCD and its interplay with promising technologies such as machine learning and personalized virtual heart models. This figure visually summarizes these goals and highlights some of the content to be covered.

Efforts to improve risk stratification for SCD, or even to reclassify risk assigned by EF, have been undertaken across all cardiac imaging modalities. Herein, we focus on the role of advanced imaging, namely cardiac magnetic resonance imaging (CMR), single-photon emission computer tomography (SPECT), and positron emission tomography (PET), and its application to risk stratification for SCD (Figure 1). We highlight unique techniques of each modality and their limitations, with particular attention to the patient subgroups that inform the utility and pathophysiology of each method (Table 1). For each study, we distinguish endpoints that are related though not interchangeable: while studies with SCD were emphasized in this review, we mention when cardiac death, all-cause mortality, or VA were the primary endpoint as they too are informative. We also describe current and future applications of machine learning (ML) in advanced imaging for SCD, from image acquisition to model construction.

**Table 1:** Comparison of imaging modalities described in this review.

**Modality**	**Sequences**	**Characteristics studied**	**Evaluation of structure**	**Evaluation of function**	**Tissue characterization**	**Cost**
Echocardiography	2D	• LVEF • Strain	++	++	+	Low
CMR	LGE T1 T2*	• Strain • Scar • Diffuse fibrosis	+++	+++	+++	Moderate
SPECT	MIBG	• Scar • Viability • Innervation	+	+	++	High
PET	FDG HED	• Scar • Viability • Innervation	++	+	++	High

+ for fair, ++ for good, and +++ for excellent.

## Cardiac magnetic resonance imaging

### Ventricular geometry and function

Chamber geometry has long been known to reflect the role of remodeling in CVD and, in turn, is associated with outcomes including VA (7, 8). Cardiac MRI has been described as the gold standard for structural and functional quantification of cardiac chambers (9, 10). Using CMR, geometry-based approaches to SCD risk stratification have emphasized these attributes, and measurements based on wall thickness have been found to distinguish patients with ICM who develop SCD (11). From a clinical standpoint, studies have suggested that at-risk patients may be inadvertently excluded from ICD as echocardiography overestimates EF when compared with CMR (12, 13). While every imaging modality is to some extent operator dependent, it is perhaps most prominently described in echocardiography as affecting interpretation (14). This highlights that LVEF thresholds may need to be individualized for different modalities, and CMR may be preferable to TTE for therapeutic decision-making in patients with intermediate-range LVEF (15). That said, EF < 35% as a selection criterion for primary prevention ICDs has routinely been critiqued for its low sensitivity and specificity in predicting VA and SCD. Furthermore, patients meeting these criteria encompass only 13% of all suffering SCD (16).

### Role of LGE in ICM and CAD

CMR with LGE has developed as a means of characterizing myocardial tissue, and its value in prognosticating cardiovascular outcomes, including SCD, has been widely reviewed and increasingly well-established (17, 18). This has been demonstrated across a wide variety of phenotypes in both patients with ICM and NICM. In ICM, heterogeneous scar, also termed gray zone, is an independent predictor of VA and SCD in a number of studies of patients with ICD or undergoing ICD implantation (19–21). A recent retrospective study among 979 patients with CAD and majority EF with > 35% showed that gray zone mass was more strongly associated with SCD than LVEF (22). The same group showed that in a mixed ICM/NICM population with ICD and cardiac resynchronization therapy, the absence of myocardial fibrosis on visual assessment virtually excluded patients at risk of VA and SCD over 7 years follow-up and among those with scar, gray zone extent added predictive value and improved net reclassification (23). Likewise, a prospective study in a mixed population of ICM and NICM patients undergoing primary prevention ICD demonstrated that the combination of low gray zone mass and low high-sensitivity C-reactive protein identified a subgroup with very low risk for VA (24). In a similar population (mixed ICM/NICM with ICD), an analysis incorporating random survival forests for model construction showed LV scar mass as well as gray zone mass by LGE were top predictors for VA and SCD (25). The same study suggested a hierarchy of risk wherein no scar was less risky than scar, larger total/core scar had higher risk than smaller scar, and larger gray zone had higher risk than smaller gray zone for the same core scar size. Additional applications of LGE, beyond presence and extent, have also been developed, including LV entropy, a measure of the distribution of pixel intensity across the myocardium (26, 27). Amongst patients with CAD and ICDs, entropy was a significant predictor for VA and SCD (27, 28). A mechanistically-minded approach in LGE has been identifying conducting channels, a nidus for VT as identified in CAD (29). A study in a mixed ICM/NICM population showed that the presence and mass of these channels were associated with the risk of VA and appropriate primary prevention ICD therapy (30).

### Role of LGE in NICM

In NICM, the role of LGE for SCD risk stratification has been frequently studied in patients with dilated cardiomyopathy (DCM) and hypertrophic cardiomyopathy (HCM) (31–33).

LGE has been used to identify higher-risk patients who fall outside the EF criterion for primary prevention ICD (34). In a multivariate model for SCD in NICM, LGE had incremental prognostic value over clinical measures, whereas EF did not (35). Among HCM patients, LGE extent has been favorably compared with clinical risk models and, in some cases, exceeded the performance of these models (36–38). To define an LGE cut-off for risk stratification, various thresholds of LGE have been described. In HCM, LGE extent >10% identified patients with SCD rates up to an order of magnitude greater than predicted with clinical risk score (39). A further analysis using serial LGE imaging in DCM illustrated that amongst patients with fibrosis progression, the majority had minimal change in EF (<5%), ergo identifying a high-risk cohort for all-cause mortality not captured by LVEF alone (40). In a case series among athletes, LGE pattern, specifically involving the lateral LV wall, was also noted to correlate with increased risk of malignant arrhythmias (41). As in ICM, LV entropy in DCM patients with ICD significantly improved a clinical model for VA, although there was only one instance of SCD among these (26). Despite these advances and its prominence in the literature, LGE has yet to be included in clinical guidelines. A likely rationale for this is that there has yet to be a completed randomized control trial using LGE for risk stratification, though several are enrolling and underway (18, 31).

Proposed diagnostic guidelines for arrhythmogenic cardiomyopathies, which carry profound and inherent risk for VA and SCD, have included LGE patterns among other criteria (42). In arrhythmogenic right ventricular cardiomyopathy/dysplasia (ARVC/D) specifically, abnormal CMR findings including LGE were associated with increased VA (43). Advances in CMR may allow for improved RV assessment facilitating earlier disease detection and ergo risk stratification (44). Similarly in myocarditis, LGE presence and extent have been associated with increased risk of major adverse cardiac events, including VA and SCD (45, 46).

### Shortcomings of LGE and alternative CMR sequences

While LGE is perhaps the most widely studied CMR method for SCD risk stratification, other CMR sequences have also been investigated, which may address certain shortcomings of LGE. An intrinsic limitation of gadolinium administration is toxicity, particularly among patients with renal insufficiency, though the degree of risk is controversial (47, 48). Fortunately, the most feared complication, nephrogenic systemic fibrosis, is exceedingly rare with modern gadolinium agents; a recent consensus statement from the American College of Radiology and the National Kidney Foundation leaves the decision for gadolinium administration in renal impairment to the clinician (49). LGE-CMR furthermore depends on several factors such as the timing of contrast injection and selection of scan parameters that can impact the interpretation of intensity values on imaging. Another limitation of LGE for risk stratification of SCD is that a subset of patients with NICM who develop VF may not have LGE on CMR (50). Several alternative measures derived from CMR have been proposed that may address some of these limitations, including native T1 mapping and extracellular volume (ECV), which reflect diffuse fibrosis, a characteristic not captured by LGE (51, 52). In a prospective study of T1 mapping and LGE assessment in participants receiving ICDs, native T1 was independently associated with VA, although performed more poorly than LGE in reclassifying participants to a low-risk group (53). Among patients with DCM, native T1 was predictive of death independent of LGE, which was present only in 27% of the study population (54). Another study in HCM patients without LGE at CMR showed an association of native T1 with SCD, though it was limited by the small number of patients (*n* = 5) reaching this endpoint (55). Less well studied is T2^*^ mapping, which combines spin-spin relaxation (T2) with magnetic field inhomogeneity to detect field distortions from the presence of materials such as iron (56). As such, it was traditionally applied to identify myocardial iron accumulation in iron storage diseases and considered arrhythmogenic in those populations (57). More recently, it has been suggested T2^*^ may add to the assessment of fibrosis, although this has not yet been well-studied (58, 59). Thus, far, a small study of the association of T2^*^ with VA in patients with HCM was negative (60). Yet another sequence, T2-weighted short-tau inversion recovery (T2w-STIR) has been used to assess myocardial edema in survivors of cardiac arrest. Presence of edema, hypothesized to represent a transient arrhythmogenic substrate, has been associated with fewer ICD shocks (61).

### Innovative uses of CMR for personalized virtual heart models to predict VA and SCD risk

A more recent development in SCD risk stratification is the use of advanced imaging to build electrophysiologic virtual heart models, which can be used to simulate arrhythmias *in silico* (62). These 3D computational models entail a biophysical approach from the cell-scale to the organ-scale and are specific to each patient's disease and resultant remodeling. Perhaps uniquely among risk stratification methodologies, virtual hearts evaluate how triggers from different locations will interact with the substrate to initiate VA, potentially providing mechanistic insights into how and VAs can develop in the patient heart (63). This approach was initially demonstrated in patients with ICM and ICDs; wherein virtual hearts were superior to clinical risk factors in predicting VA (64). This work has also been extended in a small study of patients not meeting ICD implantation criteria, distinguishing patients with VT history from those without (65). These models make use of complex patterns of imaging data such as distribution and degree of fibrosis, as well as a fusion of different pulse sequences as was done with T1 mapping in HCM (66). It is worth noting these studies have all been retrospective thus far regarding event prediction. However, virtual heart technology has recently been used to prospectively identify VT ablation targets in a small cohort, extending the utility of CMR from predicting SCD risk to guiding therapy (67).

## Nuclear imaging

### Overview

Nuclear medicine presents another advanced imaging modality that has been applied for risk stratification of SCD (68). Single-photon emission computer tomography (SPECT) is commonly used in cardiology for myocardial perfusion imaging (MPI) to assess coronary patency (69). Among patients with CAD, composite scores of fixed and reversible perfusion defects determined *via* SPECT are independently associated with SCD, including patients with EF > 35% (70, 71). These results were also reproduced in patients without significant CAD (72). Positron emission tomography (PET) provides another method of MPI, with the degree of myocardial abnormalities, such as scar, ischemia, or hibernating myocardium, associated with cardiac death (although data on PET MPI and SCD specifically is sparse) (73, 74). Both PET and SPECT can be used to characterize myocardial scar, a similar substrate to what is assessed by LGE. In an unadjusted analysis, scar extent by SPECT was associated with SCD (75). A larger study using PET among patients with EF <35% showed that scar alone, and not reversible ischemia, was significantly associated with ICD firing and SCD (76). Interestingly in the study above, the scar alone was not a significant predictor for SCD after adjustment, although it is worth noting participants in this study had a considerably higher EF (70). Combining MPI with imaging of cardiac inflammation using (18) F-fluorodeoxyglucose (FDG) has identified higher risk NICM patients independent of LVEF and clinical markers (77). Other myocardial features associated with SCD that PET and SPECT can image include sympathetic innervation and hibernating myocardium (78–80).

### Means of measuring sympathetic innervation and its significance

Assessment of innervation is conducted with radiolabeled catecholamines, most commonly iodine-123-labeled metaiodobenzylguanidine (MIBG) in SPECT though several tracers have also been studied for use with PET, most notably 11C-hydroxyephedrine (HED). Globally decreased uptake of these tracers is thought to reflect the increased sympathetic tone, which can trigger malignant arrhythmias (81). However, regional reductions in uptake can be seen in sympathetic denervation caused by myocardial ischemia and are prevalent in CAD (82). Studies, primarily undertaken amongst patients with ICM, have demonstrated both global and regional approaches for risk stratification of SCD. A prospective study of patients with HFrEF showed that reduced global uptake of MIBG was significantly associated with potentially lethal arrhythmias and cardiac death (83). Using a different measure of reuptake, another group demonstrated that abnormal MIBG washout in HFrEF was associated with SCD specifically (though this group excluded the use of beta-blockers, perhaps limiting the generalizability of the study) (84). Using PET with HED, a study among ICM patients showed regionally reduced uptake; specifically, the volume of denervated tissue was associated with SCD (85). This was similarly demonstrated with regional MIBG washout being associated with SCD (86). The role of sympathetic denervation in HFpEF and NICM continues to be explored, and studies have shown decreased MIBG uptake is associated with increased mortality and readmission. However, the relationship with SCD is unclear (87–89).

### Hibernating myocardium

Conceptually, hibernating myocardium describes regions of viable tissue with chronically reduced function and resting perfusion caused by chronic ischemia or recurrent (90). This is thought to be an arrhythmogenic substrate and has been associated with VA in porcine models (91, 92). In studies of patients with ischemic cardiomyopathy, the extent of hibernating myocardium has been associated with all-cause mortality and composite cardiac deaths (93, 94). However, in the aforementioned study of PET with HED in ICM, hibernating myocardium was not associated with SCD and was only rarely identified (85). It has been suggested that modern revascularization strategies and medical therapy may diminish the role of hibernating myocardium for risk stratification of SCD (95).

### Future potential of hybrid imaging

It is worth briefly noting that hybrid PET/MRI scanners have become commercially available relatively recently (since 2010) and brought with them a unique set of technical challenges as well as clinical possibilities (96). Thus far, PET/MRI has been applied in similar roles as its constituent modalities—in perfusion and viability studies (97). An example comparing PET and CMR in the same patient, albeit not using a hybrid scanner, demonstrates the utility of each modality. PET/MRI has in particular been investigated for diagnosis of cardiac sarcoidosis and proof-of-concept demonstrated; it is speculated that the increased quality of data gathered with PET/MRI in sarcoid patients may 1 day be used for identification of those at greater risk for VA and death (98–101). However, the role of PET/MRI in risk stratification remains hypothetical, and no study has yet described the association of any PET/MRI measure with SCD (96).

## Computed tomography

CT is presently a more accessible and generally lower cost modality than those previously mentioned. It provides higher spatial resolution and for certain purposes better temporal resolution as well. CT is perhaps most prominently used in cardiology for non-invasive assessment of the coronary arteries, the presence (or absence) of which is diagnostically significant and certainly a significant predictor of both VA and SCD (102). In terms of tissue characterization however, CT lags behind CMR, PET, and SPECT though developments are being made rapidly to improve this (103). It is also worth noting that there are patients in whom CMR may be contraindicated due to implants or foreign objects, or where ICD-related artifact is too limiting, in whom CT may provide a necessary alternative for risk stratification in the future.

CT has been used to detect fibrosis, such as by iodinated contrast enhancement (104). Scar qualification/quantification by CT has been favorably compared with LGE-CMR (105). A group recently used CT to map wall thinning to identify potential VT isthmuses in post-MI patients with a VT history, yielding 100% sensitivity when compared with gold-standard EP studies although with 50% positive predictive value (106). Similarly, regions of myocardial fat deposition as characterized by CT have been associated with VT circuit sites in patients with history of VA (107). CT has also been used to identify areas of lipomatous metaplasia after infarction which are associated with VA in experimental models (108). As with the previously mentioned modalities, CT has also been used for development of virtual heart models (109).

## The emerging role of machine learning and artificial intelligence

### Machine learning in image acquisition

In recent years, machine learning techniques have seen increasing integration within medicine, particularly as applied in imaging (110). This has specifically included applications across numerous modalities in cardiology, including CMR and nuclear imaging (111). The value of ML in the risk stratification of SCD can be appreciated at multiple stages of the imaging pipeline (Table 2). ML has been used to enhance image processing on a granular level, such as by voxel denoising. In time-intensive modalities such as MRI, this has been shown to allow for a significant reduction of acquisition time while preserving quantitative metrics such as demonstrated in brain imaging of cerebral blood flow (112). On a more experimental level, ML has been applied in low-field MRI to address reconstruction using noisy data, which may increase CMR availability in less resource-rich environments (124, 125). Finally, as an analog in PET, the clinical utility of dynamic scans required for more rapid imaging and low count protocols minimizing radiation exposure in young patients is limited by poorer image quality (113). While hardware advances provide one means of addressing this challenge, a likely more cost-effective pathway is the utilization of ML to improve the quality of the reconstructed image (126). In parallel to improving image quality, ML has been used to lower the computational burden of contemporary post-processing techniques such as scatter correction in PET (114, 115). Another forward-looking application of ML is in attenuation and scatter correction for PET-only, SPECT-only, or hybrid PET/MRI imaging, as this is conventionally accomplished with simultaneous CT (116). This may increase the accessibility and capability of advanced imaging and allow for a broader range of patients to undergo risk stratification with these modalities.

**Table 2:** Incorporation of machine learning into advanced imaging.

**Imaging steps**	**Role of ML**	**Examples of applications**
Acquisition	• Increasing acquisition speed • Decreasing radiation exposure	• Voxel denoising in CMR (112) • Implementing low count (radiation) protocols for PET (113)
Processing	• Reducing computation burden • Automating labor-intensive analysis • Standardization	• Scatter correction (114, 115) • Synthetic CT (applied to PET, SPECT, and PET/MRI) (116) • Automated scar quantification (117) • Common algorithm for segmentation across multiple centers (118)
Feature extraction	• Generating novel features, texture analysis • Investigation of hypothetical markers	• ML-derived scar heterogeneity (119) • Generating hundreds of features from individual sequences (120)
Model construction	• Advanced analytics • Dimension reduction; identifying significant markers • Data synthesis	• Applying random survival forests (121) • Combining non-simultaneous PET, MRI modalities (122) • Unified analysis using EMR (123)

### Machine learning in image processing

Perhaps more widely reported are clinician-facing advances in image analysis and interpretation using ML, described by the overarching term “computer vision” to include any automated interpretation of images (127). ML-based algorithms have been available in commercial software for segmentation for some time now. They are widely used for automated quantification of structure and function, for example, ventricular volumes and EF (128). Some of these data are, as previously mentioned, used in models for the prediction of SCD. However, automation has extended to tissue characterization as well. LGE segmentation, when performed manually, can be a labor-intensive process requiring significant training to mitigate subjectivity and inter-operator variability (129).

Nonetheless, manual segmentation has previously been considered the gold standard for accuracy, especially when compared with techniques based on older ML algorithms (130). More recent studies have demonstrated the feasibility of novel approaches, such as deep convolutional neural networks, for automated scar quantification approaching manual segmentation in subsets of patients such as those with ICM and HCM (117, 131). This has also shown promise in multicenter datasets, potentially addressing the challenge of practice standardization (118, 132).

### Machine learning for feature extraction

Sequential to obtaining imaging measures is its interpretation and application to predicting cardiovascular endpoints. Techniques such as texture analysis may be applied to pre-existing LGE segmentation to obtain features beyond quantitative scar burden, such as measures of heterogeneity and shape, which have been proven valuable in conventional workflows (119, 133). Numerous extracted features and varying ML models have been evaluated for this role; in other words, ML can be used to identify novel predictors and implement novel prediction methods (134, 135). However, these extracted features are typically unrecognizable to the human eye, especially when obtained from composite, multistep analyses necessitating further selection to identify covariates with the strongest predictive value (119, 136). For example, in a cohort of patients with DCM, principal component analysis was applied across ventricular geometric models to derive shape-based features, which were integrated into a score that was shown to be independently associated with composite VA and SCD (137). Another study generated 608 features from LGE and T2 sequences in Takotsubo patients to predict outcomes, including MI and death (120). ML was also used for dimensionality reduction in this study, which interestingly resulted in all LGE-derived features being discarded. Along these lines, a recent study used an unsupervised, deep learning approach on cine CMR among ICM patients to derive cardiac features that were then used as inputs in a separate deep neural network that successfully predicted VA risk (138). The inverse approach, using ML to generate pre-defined features, is also appreciable in recent literature. For example, ML-derived measures based on LGE, such as scar complexity, were associated with VA in a cohort where entropy was not a significant predictor (139).

### Innovating the role of advanced imaging with machine learning

In the future, machine learning may be integrated into the risk stratification workflow from the point of image acquisition to patient-facing risk prediction models, and there are studies demonstrating this in principle. One example is incorporating manual measurements and segmentation with using ML for feature extraction and statistical model-building (134). Sophisticated virtual heart simulations incorporating both MRI and PET data, then using ML to synthesize imaging and clinical data for SCD prediction, have been shown to outperform existing risk models (122). This approach is not specific to VA or SCD outcomes, and it has also been used to predict improvement in EF after cardiac resynchronization therapy (140). Because of the sheer volume of data available to the clinician from the electronic medical record and conglomerate imaging, it seems increasingly likely that ML will play a central role in the fusion of data of different sources and risk modeling (123, 141–143).

## Conclusion

As described in this review, there are numerous promising applications of advanced imaging to identify patients at risk of SCD. Some of the above techniques and sequences may likely be incorporated into clinical guidelines and ultimately into regular practice in the coming years. Machine learning may enable advances democratizing advanced imaging for at-risk patients, as has been achieved with CT screening in smoking. Physicians and cardiologists of the future will likely have a wide variety of complementary imaging modalities and analytic tools to identify SCD risk amongst different patient populations optimally.

## Author contributions

EX and JC contributed to the conception, tables, and design of the manuscript. EX, ESu, ESa, and JC contributed to creation and editing of figures. All authors agree to be accountable for the content of the work. All authors contributed to the drafting, editing, and review of the manuscript. All authors contributed to the article and approved the submitted version.

## Funding

The authors acknowledge funding from NIH (NIH/NHLBI R01HL103812 and R01HL132181 to KW and R01HL142496 and R01HL124893 to NT), Leducq Foundation (NT), and AHA (Predoctoral Fellowship to Esu).

## Conflict of interest

The authors declare that the research was conducted in the absence of any commercial or financial relationships that could be construed as a potential conflict of interest.

## Publisher's note

All claims expressed in this article are solely those of the authors and do not necessarily represent those of their affiliated organizations, or those of the publisher, the editors and the reviewers. Any product that may be evaluated in this article, or claim that may be made by its manufacturer, is not guaranteed or endorsed by the publisher.
